# Airway Exposure to Polyethyleneimine Nanoparticles Induces Type 2 Immunity by a Mechanism Involving Oxidative Stress and ATP Release

**DOI:** 10.3390/ijms22169071

**Published:** 2021-08-23

**Authors:** Yotesawee Srisomboon, Noriyuki Ohkura, Koji Iijima, Takao Kobayashi, Peter J. Maniak, Hirohito Kita, Scott M. O’Grady

**Affiliations:** 1 Department of Animal Science, University of Minnesota, St. Paul, MN 55108, USA; ysrisomb@umn.edu (Y.S.); mania002@umn.edu (P.J.M.); 2 Division of Allergic Diseases, Mayo Clinic, Scottsdale, AZ 85259, USA; n-ohkura@med.kanazawa-u.ac.jp (N.O.); Iijima.Koji@mayo.edu (K.I.); Kobayashi.Takao@mayo.edu (T.K.)

**Keywords:** allergic inflammation, purinergic signaling, IL-33, Th2 cytokines, intracellular Ca^2+^

## Abstract

Polyethyleneimine (PEI) induced immune responses were investigated in human bronchial epithelial (hBE) cells and mice. PEI rapidly induced ATP release from hBE cells and pretreatment with glutathione (GSH) blocked the response. PEI activated two conductive pathways, VDAC-1 and pannexin 1, which completely accounted for ATP efflux across the plasma membrane. Moreover, PEI increased intracellular Ca^2+^ concentration ([Ca^2+^]_i_), which was reduced by the pannexin 1 inhibitor, ^10^Panx (50 μM), the VDAC-1 inhibitor, DIDS (100 μM), and was nearly abolished by pretreatment with GSH (5 mM). The increase in [Ca^2+^]_i_ involved Ca^2+^ uptake through two pathways, one blocked by oxidized ATP (oATP, 300 μM) and another that was blocked by the TRPV-1 antagonist A784168 (100 nM). PEI stimulation also increased IL-33 mRNA expression and protein secretion. In vivo experiments showed that acute (4.5 h) PEI exposure stimulated secretion of Th2 cytokines (IL-5 and IL-13) into bronchoalveolar lavage (BAL) fluid. Conjugation of PEI with ovalbumin also induced eosinophil recruitment and secretion of IL-5 and IL-13 into BAL fluid, which was inhibited in IL-33 receptor (ST2) deficient mice. In conclusion, PEI-induced oxidative stress stimulated type 2 immune responses by activating ATP-dependent Ca^2+^ uptake leading to IL-33 secretion, similar to allergens derived from Alternaria.

## 1. Introduction

The commercial production of engineered nanomaterials (ENMs) is a steadily growing industry with increasing potential to impact human health as exposure to consumer products containing ENMs becomes more prevalent [1,2,3,4,5]. Studies addressing the safety of nanoscale particles indicate that their physicochemical properties (small size (<100 nm), chemical composition, electronic charge, large surface area to mass ratio, surface coating, potential for generating reactive oxygen species, and deep penetration) are capable of producing adverse effects on lung function including exacerbation of asthma and sensitization to common allergens [5,6,7,8,9]. Due to their small size, inhaled ENMs become widely dispersed throughout the airways and alveoli, where uptake into various cell types creates the potential for altering normal cell and tissue function [5,6,10,11]. Moreover, ENMs have been shown to induce cytotoxicity associated with Ca^2+^ uptake, mitochondrial depolarization, and cell membrane damage that can trigger inflammation [5,12].

Certain types of ENMs have important biomedical applications, serving as excellent carrier molecules that are capable of encapsulating drugs, nucleic acids and contrast agents [13,14]. ENMs derived from branched polyethylenimine (PEI; 25 kDa) were initially studied and used as non-viral DNA carriers for gene therapy [15]. Subsequently, applications were extended to other nucleic acids including small RNAs, siRNAs, miRNAs, anti-miRNAs, and hammerhead ribozymes [16]. PEI polyplexes are often described as the gold standard of gene transfection reagents since they exhibit the highest transfection efficiency among non-viral vectors when used under serum-free conditions [17]. PEI possesses a large number of positively charged amine groups, which enables electrostatic condensation with negatively charged molecules such as nucleic acids [14,18]. These amine groups also absorb protons (the so-called proton sponge effect) which protects DNA and RNA from nuclease activity within the acidic environment of endosomal/lysosomal compartments, ensuring escape of undamaged DNA or RNA into the cytoplasm [16]. When PEI is used in excess during complexation and condensation reactions, PEI-nucleic acid polyplexes are formed that possess a net positive charge. This positive zeta potential enables electrostatic interactions with specific negatively charged constituents within the plasma membrane, including heparin sulfate and proteoglycans, which facilitates uptake across the cell membrane by endocytic, pinocytotic or phagocytic mechanisms [16]. Notably, cytotoxicity appears to be associated with the positive charge of PEI polyplexes.

In response to the global pandemic caused by SARS-CoV-2 (COVID19), pharmaceutical companies and academic research institutions have rushed to create vaccines ranging from conventional viral and protein based designs to pioneering mRNA-based vaccines [19,20,21,22]. At this time, all clinically used COVID19 mRNA vaccines are delivered by lipid-based nanoparticles that contain: (1) ionizable lipids that form complexes with mRNA, (2) phospholipids and cholesterol to facilitate formation and stabilization of the particle, and (3) PEGylated lipids to minimize non-specific interactions [23]. However, other types of mRNA delivery vehicles have also been developed based on polymer or polymer/lipid hybrid formulations that have been used for in vivo antigen delivery [20]. Low molecular weight (2 kDa), PEI-based polyplexes conjugated to β-cyclodextrin (β-CD) represents an example of a polymer type nanoparticle, which is efficiently taken up by cells into the endosomal compartment where the mRNA separates from the β-CD/PEI conjugate and escapes into the cytoplasm [24]. In a previous study by Li et al., (2016) a β-CD/PEI conjugate delivery system was used to immunize BALB/c mice intranasally with HIV gp120 mRNA [25]. The authors found that these nanoparticles effectively penetrated the nasal epithelial barrier by reversibly opening tight junctions, allowing for greater paracellular delivery of mRNA. The vaccination triggered a strong mucosal anti-HIV gp120 immune response with a balanced Th1/Th2/Th17-type cytokine profile. β-CD/PEI conjugate platforms have also been used for in vivo transfection of mRNAs for the model antigen, ovalbumin (OVA), subcutaneously, intradermally, and intramuscularly into BALB/c mice. Intramuscular (IM) and intradermal (ID) vaccination generated a mixed Th1/Th2 type immune response, wherein IM administration yielded a tendency towards Th2-type immunity, while ID vaccination evoked a Th1 response [25]. In contrast, subcutaneous vaccination failed to generate a detectable IgG response. Thus, these studies demonstrate the potential of using polymer-based, PEI containing delivery systems for transfection with mRNA-type vaccines.

In the present study, we investigated the underlying mechanisms leading to type 2 inflammation associated with PEI exposure. We tested the hypothesis that treatment with PEI induces type 2 immunity in a manner similar to certain environmental allergens such as those derived from the fungus Alternaria alternata. We measured ATP release, Ca^2+^ uptake and IL-33 secretion induced by PEI in human airway epithelial cells and compared the data to previously published results and new data obtained after exposure to Alternaria. We also examined the in vivo effects of PEI on acute induction of type 2 cytokine secretion into bronchoalveolar lavage (BAL) fluid and the effects of combined ovalbumin and PEI challenge on immune responses in control and IL-33 receptor (ST2) deficient mice. The results demonstrated that both common and distinct mechanisms for ATP release and Ca^2+^ uptake were activated by PEI compared to Alternaria. Furthermore, PEI acutely increased IL-33, IL-5, and IL-13 secretion into the airway lumen and PEI conjugated ovalbumin produced a type 2 immune response that was significantly reduced in ST2 deficient mice.

## 2. Results

**Figure 1:**
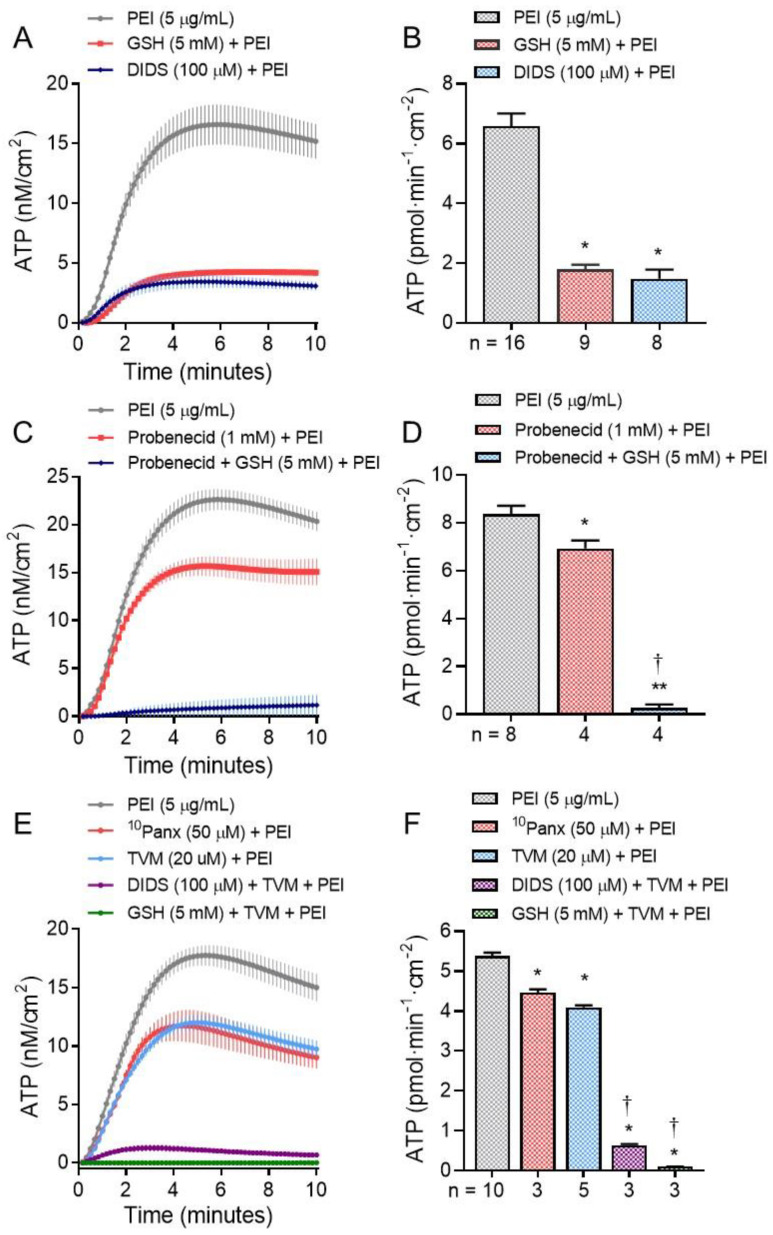
Effects of PEI on the kinetics of ATP release from hBE cells. (**A**). Time course measurements of ATP release following exposure to PEI (5 μg/mL) alone (*n* = 16) or after pretreatment (1 h) with glutathione (GSH: 5 mM; *n* = 9) or DIDS (100 μM; *n* = 8). (**B**). Initial rates of ATP release measured between 0.5 and 2.0 min after exposure. Data points were fit using linear regression analysis (PEI alone, *n* = 16; GSH + PEI, *n* = 9; DIDS + PEI, *n* = 8, * *p* < 0.0001, ** *p* < 0.0264 compared to PEI alone; ^†^ *p* < 0.0001 comparing Probenecid + PEI with Probenecid + GSH + PEI). Initial rates of ATP release were determined from the slopes and plotted in the bar graph. GSH + PEI and DIDS + PEI conditions were significantly different from PEI alone; Brown-Forsythe and Welch ANOVA with Dunnett’s T3 posttest. (**C**). Effects of probenecid (1 mM) and probenecid + GSH (5 mM) on PEI-induced ATP release (PEI (5 μg/mL) alone, *n* = 8; Probenecid (1 mM) + PEI, *n* = 4; Probenecid + GSH (5 mM) + PEI, *n* = 4). (**D**). Initial rates of ATP release calculated from data generated in Figure 1C. Data points were fit using linear regression analysis (PEI (5 μg/mL) alone, *n* = 8; Probenecid (1 mM) + PEI, *n* = 4; Probenecid + GSH (5 mM) + PEI, *n* = 8). Initial rates of ATP release were determined from the slopes and plotted in the bar graph. Probenecid + PEI and Probenecid + GSH + PEI conditions were significantly different from PEI alone (* *p* < 0.0001) and GSH (5 mM) + Probenecid was significantly different from Probenecid (1 mM) + PEI (^†^ *p* < 0.0001); ANOVA with Tukey’s posttest. (**E**). Effects of pannexin 1 inhibitors, ^10^Panx peptide (50 μM; *n* = 3) and Trovafloxacin mesylate (TVM, 20 μM; *n* = 5) on PEI (5 μg/mL; *n* = 10)-induced ATP release. GSH (5 mM; *n* = 3) and DIDS (100 μM; *n* = 3) produced additive effects when co-administered with TVM (20 μM), which abolished ATP release. (**F**). Initial rates of ATP release calculated from data shown in Figure 1E. Data points were fit using linear regression analysis (PEI (5 μg/mL) alone, *n* = 10; ^10^Panx peptide (50 μM; *n* = 3), TVM (20 μM; *n* = 5), DIDS (100 μM) + TVM + PEI, *n* = 3 and GSH (5 mM) + TVM + PEI, *n* = 3). Initial rates of ATP release were determined from the slopes and plotted in the bar graph. ^10^Panx + PEI, Trovafloxacin mesylate (TVM) + PEI, DIDS + TVM + PEI and GSH + TVM + PEI conditions were significantly different from PEI alone (* *p* < 0.0001) or from TVM + PEI; ANOVA (^†^ *p* < 0.0001) with Tukey’s posttest.

Previous studies showed that human bronchial epithelial (HBE) cells exhibit a rapid (within minutes) and sustained release of ATP following exposure to Alternaria [26]. To determine if PEI produces a similar response, kinetic measurements of ATP release using a luciferin/luciferase-based photon emission assay were performed (Figure 1; [27,28]). PEI (5 μg/mL) induced a sustained increase in extracellular ATP and pretreatment with the reactive oxygen species (ROS) scavenger, glutathione (5 mM), or with DIDS (100 μM) an inhibitor of the voltage-dependent anion channel (VDAC-1), reduced the PEI-stimulated increase in extracellular ATP by ~75% and 79%, respectively (Figure 1A,C,E). Initial rates of ATP release were determined from linear regression analysis of the ATP kinetic data (Figure 1A) between 0.5–2.0 min for PEI (6.58 ± 0.43), GSH + PEI (1.79 * ± 0.16) and DIDS + PEI (1.48 * ± 0.30) (Figure 1B). GSH and DIDS significantly inhibited the initial rate of release by ~73% and ~78%, respectively (Figure 1B; * *p* < 0.0001 compared to PEI alone). GSH + PEI and DIDS + PEI treatment conditions were not significantly different. Figure 1C shows the effects of probenecid (1 mM), a previously characterized inhibitor of pannexin 1 channels [29], on PEI-induced ATP release. Probenecid inhibited the PEI-evoked steady state increase in extracellular ATP by ~31% and when cells were pretreated with both GSH and probenecid, an additive response was observed, amounting to ~96% inhibition of the total ATP response. The initial rates of ATP release (Figure 1D) were PEI (5 μg/mL) = 8.37 ± 0.34, probenecid (1 mM) + PEI = 6.93 ** ± 0.33 (~17% inhibition of the ATP response; ** *p* < 0.0264 compared to PEI alone), and Probenecid (1 mM) + GSH (5 mM) + PEI = 0.26 ± 0.15 (~97% inhibition of the ATP response; * *p* < 0.0001 compared to PEI alone and Probenecid + PEI; ^†^ *p* < 0.0001 comparing Probenecid + PEI with Probenecid + GSH + PEI). Two additional inhibitors of pannexin-1, including the selective peptide blocker ^10^Panx (50 μM) and the antibiotic, trovafloxacin mesylate (TVM; 20 μM) inhibited the PEI (5 μg/mL)-induced increase in extracellular ATP by 32 and 38%, respectively [30,31]. When TVM (20 μM) was added in combination with DIDS (100 μM) or GSH (5 mM), the PEI-stimulated increase in extracellular ATP was inhibited by ~95% and ~99%, respectively (Figure 1E). Figure 1F shows the results of initial rate measurements derived from linear regression analysis of the data presented in Figure 1E where PEI = 5.38 ± 0.27, ^10^Panx + PEI = 4.47 ** ± 0.13 (** *p* < 0001 compared to PEI alone) and TVM + PEI = 4.10 * ± 0.11 (* *p* < 0.0001 compared to PEI alone). DIDS and GSH produced additive responses that essentially abolished PEI-evoked ATP release (TVM + DIDS + PEI = 0.64 * ± 0.05 and TVM + GSH + PEI = 0.001 * ± 0.0003 where * *p* < 0.0001 compared to PEI alone and ^†^ *p* < 0.0001 when compared with TVM + PEI).

**Figure 2:**
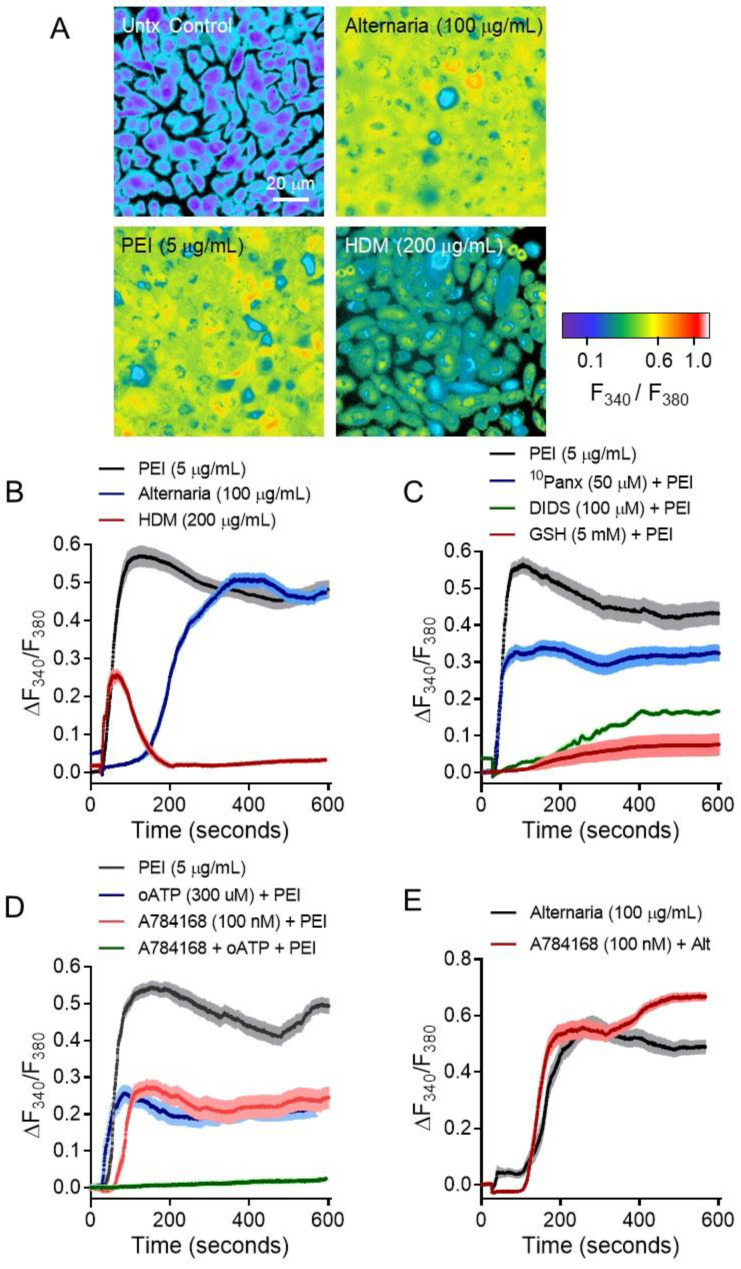
Allergen and PEI induced increases in [Ca^2+^]_i_. (**A**). Pseudo-color images of hBE cells loaded with the ratiometric Ca^2+^ indicator, Fura-2. The resting level of intracellular [Ca^2+^] is shown in the untreated (Untx) control image. After exposure to PEI (5 μg/mL) or Alternaria (100 μg/mL), the increase in [Ca^2+^] is indicated by a change in color from blue to yellow/orange. In cells treated with 200 μg/mL HDM, the peak Ca^2+^ response was less than observed with PEI or Alternaria. (**B**). Comparison of the kinetics of PEI (5 μg/mL; *n* = 36), Alternaria (100 μg/mL; *n* = 36) and HDM (200 μg/mL; *n* = 36)—evoked Ca^2+^ responses. (**C**). Inhibitors of pannexin 1 (^10^Panx, 50 μM; *n* = 36), VDAC-1 (DIDS, 100 μM: *n* = 36) and oxidative stress (GSH, 5 mM; *n* = 36), which block ATP release also reduced PEI (5 μg/mL)-induced increases in [Ca^2+^]_i_. (**D**). The selective TRPV1 antagonist (A784168, 100 nM, *n* = 36) and the P2X-receptor antagonist (oATP, 300 μM; *n* = 36) inhibited ~50% of the PEI-induced Ca^2+^ response. When hBE cells were pretreated with both antagonists, the PEI-stimulated Ca^2+^ response was completely blocked. (**E**). In contrast, the TRPV1 antagonist A784168 (100 nM; *n* = 36) does not inhibit Alternaria-evoked increases in [Ca^2+^]_i_.

hBE cell exposure to PEI induced a sustained increase in intracellular [Ca^2+^] ([Ca^2+^]_i_) similar to Alternaria. Figure 2A shows images of hBE cells loaded with the ratiometric Ca^2+^-sensing indicator Fura 2 after exposure to Alternaria (100 μg/mL), PEI (5 μg/mL) or house dust mite extract (HDM, 200 μg/mL) and Figure 2B shows the kinetics of the Ca^2+^ response. Removal of Ca^2+^ from the extracellular solution abolished the increase in [Ca^2+^]_i_ induced by PEI (steady state ΔF340/F380 following PEI exposure = 0.0036 ± 0.006, which was not significantly different from basal [Ca^2+^]_i_). This result indicated that the PEI-stimulated increase in [Ca^2+^]_i_ was due to an increase in Ca^2+^ uptake across the plasma membrane. The PEI-evoked increase in [Ca^2+^]_i_ was more rapid than that produced by Alternaria, but similar to the more transient Ca^2+^ response induced by HDM. Figure 2C shows that inhibitors of ATP release (^10^Panx (50 μM), DIDS (100 μM) and GSH (5 mM)) reduced PEI-stimulated Ca^2+^ uptake by amounts corresponding to their inhibitory effects on PEI-stimulated ATP release.

In a previous study, *Alternaria*-induced Ca^2+^ uptake was inhibited by oATP, suggesting a possible role for P2X receptors in facilitating Ca^2+^ entry into the cell [30]. In Figure 2D, treatment of hBE cells with oATP (300 μM) inhibited PEI-evoked Ca^2+^ uptake by ~50%. Similarly, treatment with the selective TRPV1 antagonist A784168 (100 nM) also inhibited ~50% of the PEI-stimulated Ca^2+^ response, and when both oATP and A784168 were added in combination, PEI-induced Ca^2+^ uptake was completely blocked. In contrast, 100 nM A784168 had no significant effect on the Alternaria-induced increase in [Ca^2+^]_i_ (Figure 2E). 

**Figure 3:**
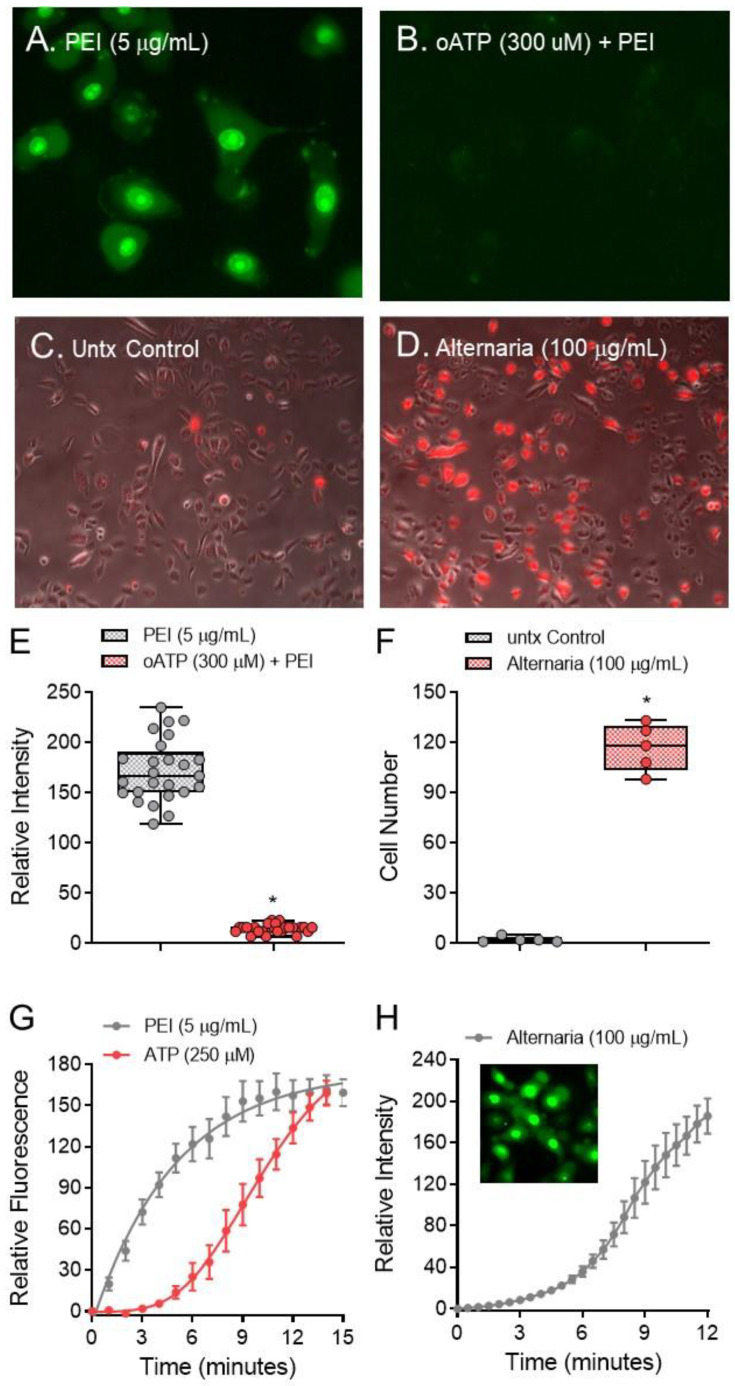
Yo Pro-1 and rhodamine uptake into hBE cells following treatment with PEI (5 μg/mL) and Alternaria (100 μg/mL). (**A**). Yo Pro-1 fluorescence detected in hBE cells after exposure to PEI for 15 min. (**B**). Pretreatment with oATP (300 μM) blocks PEI-induced Yo Pro-1 uptake. (**C**). Control experiment showing that hBE cells do not exhibit rhodamine uptake. (**D**). Alternaria (100 μg/mL) stimulates rhodamine influx. (**E**). PEI (5 μg/mL; *n* = 25) induced YoPro-1 uptake measured in units of relative fluorescence intensity is blocked by oATP (300 μM; *n* = 25; * *p* < 0.0001). (**F**). Rhodamine uptake into a population of 150 hBE cells before and after Alternaria (100 μg/mL; *n* = 5 experiments) exposure (* *p* < 0.0001). (**G**). Kinetics of PEI (5 μg/mL; *n* = 9) and ATP (250 μM; *n* = 10)-induced Yo Pro-1 uptake. (**H**). Kinetics of Alternaria (100 μg/mL; *n* = 5) induced Yo Pro-1 uptake.

Stimulation of hBE cells with PEI produced an increase in uptake of the fluorescent cationic dye Yo Pro-1 (mw 629). Figure 3A shows Yo Pro-1 labeling of both the nucleus and cytoplasm of hBE cells after exposure to PEI (5 μg/mL) for 15 min. Pretreatment with oATP (300 μM) abolished PEI-induced Yo Pro-1 uptake (Figure 3B). Quantitation of the oATP effect is shown in Figure 3E (* *p* < 0.0001, *n* = 25). Figure 3C,D show rhodamine B (mw 479) labeling of hBE cells before and after Alternaria stimulation. In the absence of Alternaria, the cell membrane was essentially impermeable to rhodamine B. However, Alternaria (100 μg/mL) exposure for 15 min induced rhodamine B uptake into most of the HBE cells. A quantitative comparison between the number of rhodamine B-labeled cells before and after Alternaria exposure is shown in Figure 3F (* *p* < 0.0001, *n* = 5). The kinetics of PEI and ATP-induced Yo Pro-1 uptake are shown in Figure 3G. Note that the PEI response is more rapid than ATP and Alternaria (100 μg/mL) on Yo Pro-1 uptake shown in Figure 3H. 

**Figure 4:**
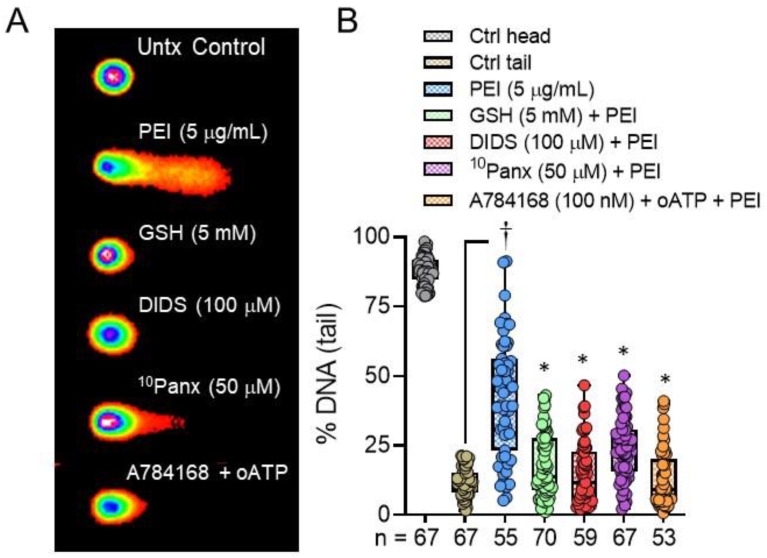
Results of comet assays showing that PEI (5 μg/mL) causes DNA fragmentation. (**A**). Images showing PEI induced comets before and after pretreatment with the oxidant scavenger GSH (5 mM), two inhibitors of ATP release (DIDS (100 μM) and ^10^Panx (50 μM)) and a combination of inhibitors that block Ca^2+^ uptake (oATP (300 μM) and A784168 (100 nM)). (**B**). Measurements of % DNA present in the tails of comets exposed to PEI (5 μg/mL; *n* = 55) before (^†^ *p* < 0.0001) and after pretreatment with GSH (5 mM; *n* = 70), DIDS (100 μM; *n* = 59), ^10^Panx (50 μM; *n* = 67) and oATP (300 μM) + A784168 (100 nM), *n* = 53 (* *p* < 0.0001). The Brown-Forsythe and Welch ANOVA followed by Dunnett’s T3 multiple comparison’s test was used to determine significant differences between groups.

PEI (5 μg/mL; *n* = 55) also caused DNA fragmentation as revealed in comet assays that show significant (^†^ *p* < 0.0001) tail formation following exposure for 30 min (Figure 4). If cells were pretreated (1 h) with the ROS scavenger glutathione (5 mM; *n* = 70) or with inhibitors of ATP release (DIDS (100 μM; *n* = 59) or ^10^Panx (50 μM; *n* = 67), DNA fragmentation was significantly (* *p* < 0.0001) blocked. Similarly, when PEI (5 μg/mL) stimulated Ca^2+^ uptake is blocked by combined pretreatment with oATP (300 μM) and A784168 (100 nM), DNA damage was also inhibited (* *p* < 0.0001; *n* = 53).

**Figure 5:**
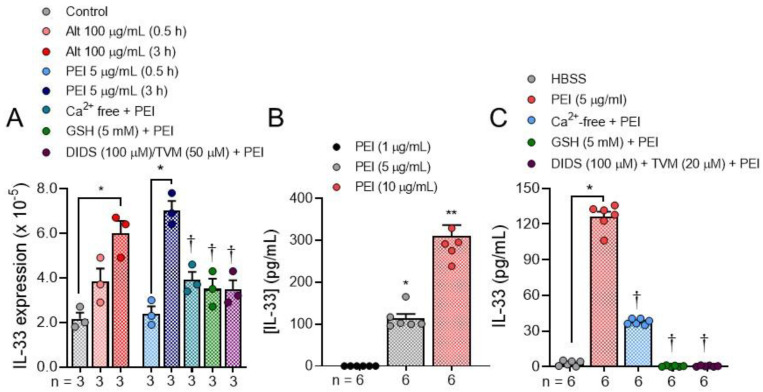
PEI stimulates IL-33 mRNA expression and secretion. (**A**). Exposure of immortalized hBE cells transfected with full length human IL-33 gene (GET-33 cells) to Alternaria (Alt) 100 μg/mL for 0.5 and 3 h resulted in a significant (* *p* < 0.0001) increase in IL-33 mRNA expression after 3 h. A similar increase was observed following exposure to PEI (5 μg/mL) at 3 h. Pretreatment with GSH (5 mM) or removal of extracellular [Ca^2+^] (Ca^2+^-free conditions) or inhibition of ATP release with both DIDS (100 μM) and TVM (20 μM) significantly blocked the PEI-induced increase in IL-33 mRNA expression (^†^ *p* < 0.0008). (**B**). Treatment of GET-33 cells with PEI for 1 h produced a concentration-dependent increase in IL-33 secretion (* *p* < 0.0002; ** *p* < 0.0001). (**C**). PEI (5 μg/mL) induced IL-33 secretion (* *p* < 0.0001) was inhibited (^†^ *p* < 0.0001) under; (i) Ca^2+^-free conditions, (ii) when oxidative stress was blocked by pretreatment with GSH (5 mM) and (iii) when ATP release was completely inhibited using DIDS (100 μM) + TVM (20 μM). Multiple comparisons were made using a one-way ANOVA followed by Tukey’s posttest for each figure.

Figure 5 shows that PEI, like Alternaria, increases IL-33 mRNA expression and protein secretion. IL-33 is a cytokine known to be stored in the nucleus of bronchial epithelial cells [32,33,34]. Allergen exposure induces proteolytic processing and secretion of IL-33 into the extracellular fluid, where it binds to ST2 receptors expressed by immune cells to stimulate expression and release of Th2 cytokines, including IL-5 and IL-13 [35,36]. The increase in IL-33 mRNA expression was significant within 3 h following exposure to PEI (* *p* < 0.0001). Furthermore, increased expression of IL-33 mRNA was blocked (^†^ *p* < 0.0008) under conditions where oxidative stress was inhibited by pretreatment with 5 mM GSH or when the PEI-induced increase in intracellular [Ca^2+^] was blocked by removal of extracellular Ca^2+^ or when ATP release was inhibited with both DIDS (100 μM) and TVM (20 μM) (Figure 5A). PEI also induced a concentration-dependent increase in IL-33 secretion from GET33 cells (* *p* = 0.0002; ** *p* < 0.0001; Figure 5B), which was inhibited (^†^ *p* < 0.0001) by the same pretreatment conditions that also reduced IL-33 mRNA expression (Figure 5C).

**Figure 6:**
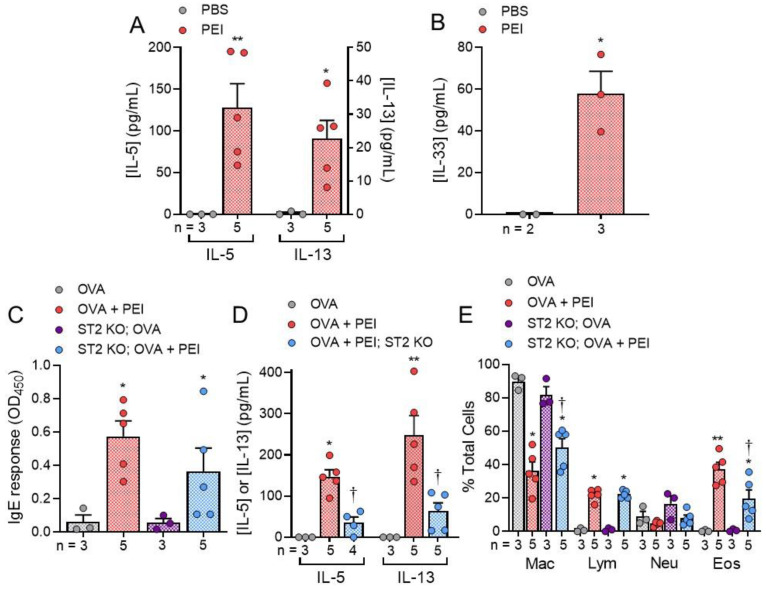
In vivo experiments showing an increase in type 2 immune responses following exposure to PEI alone or PEI-OVA polyplexes. (**A**). Intranasal PEI stimulates IL5 and IL-13 secretion into BAL fluid (* *p* = 0.0260; ** *p* < 0.0113). (**B**). PEI (12.5 μg/mL) also increased IL-33 concentration in BAL samples (* *p* = 0.0324). (**C**). Effects of OVA and OVA + PEI on the level of IgE in plasma (* *p* < 0.03). (**D**). Intranasal administration of PEI + OVA increases IL-5 and IL-13 secretion, which was blocked in ST2 deficient mice (* *p* = 0.0017; ** *p* = 0.0078). (**E**). BAL immune cell counts following stimulation with 5 μg/mL PEI exhibited increased numbers of lymphocytes and eosinophils but not neutrophils (Mac: * *p* < 0.0001 compared to OVA alone; ^†^ *p* = 0.0073 compared to ST2 KO; OVA; Lym: * *p* < 0.0001 compared to OVA alove or ST2 KO; OVA). The increase (** *p* = 0.0003 comparing OVA alone to OVA + PEI and * *p* = 0.0424 comparing ST2 KO; OVA to ST2 KO; OVA + PEI) in eosinophils was reduced in ST2 deficient mice (Eos: ^†^ *p* < 0.0290 comparing OVA + PEI to ST2 KO; OVA + PEI). Unpaired *t*-tests were used in A and, B, and a one-way ANOVA followed by Tukey’s multiple comparison’s posttest was used in (**C**–**E**).

In vivo experiments involving intranasal administration of PEI into mouse airways revealed increased levels of IL-5, IL-13 (** *p* = 0.0113; * *p* = 0.0260) and IL-33 (* *p* = 0.0324) in BAL fluid after 4.5 h (Figure 6A,B). Intranasal challenge with PEI complexed with the model antigen ovalbumin (OVA) also produced significant increases (* *p* = 0.03, comparing OVA with OVA + PEI and ST2 KO; OVA with ST2 KO; OVA + PEI) in plasma IgE levels that were not significantly reduced in ST2 deficient mice (Figure 6C). Although IL-33 is known to induce follicular helper T cells, it is important to note that other proinflammatory molecules/pathways can also drive IgE secretion by these cells. Thus, the IgE response is less dependent on the IL-33/ST2 pathway [37]. In addition, mice previously exposed to OVA+PEI produced more IL-5 and IL-13 in BAL fluids as compared to those exposed to OVA alone when they were challenged with OVA. When this experiment was repeated, using ST2 deficient mice, BAL levels of IL-5 and IL-13 were significantly reduced compared to control mice (Figure 6D). Moreover, analysis of immune cell recruitment into BAL fluid samples following OVA challenge of mice that had been exposed to OVA+PEI showed significant increases in lymphocytes and eosinophils, with no significant recruitment of neutrophils. However, ST2 deficient mice exhibited a significantly lower amount of eosinophil recruitment into BAL fluid relative to control mice (Figure 6E). These results are consistent with induction of type 2 immunity following challenge with PEI alone or when challenged with PEI-OVA complexes. 

## 3. Discussion

Previous studies have demonstrated that allergens derived from Alternaria alternata, house dust mites (HDM) and cockroaches stimulate ATP release from airway epithelial cells, which functions as a critical early step in the processing, nuclear mobilization, and secretion of IL-33 [27,38,39,40]. In the case of Alternaria allergen exposure, two mechanisms of ATP release have been described: a conductive pathway involving VDAC-1 and exocytosis of ATP contained within membrane vesicles [28,41]. ROS scavengers including GSH and N-acetylcysteine (NAC) inhibited Alternaria-evoked ATP release mediated by VDAC-1, indicating a role for oxidative stress in activating the channel [41]. The disulfonic stilbene compound DIDS also blocked VDAC-1 mediated ATP release to the same extent as GSH and NAC [41]. In the present study, GSH and DIDS each inhibited approximately 75% of the initial rate of ATP release induced by PEI, consistent with inhibition of VDAC-1-dependent ATP release, previously observed in response to Alternaria exposure. Pretreatment of hBE cells with three known inhibitors of pannexin-1, inhibited the remaining PEI-stimulated, GSH and DIDS-insensitive ATP release. Pannexin-1 channels are well known for conducting ATP across the plasma membrane and earlier studies have shown that they are activated by variety of stimuli including increases in extracellular [K^+^], membrane depolarization, mechanical stimulation, increases in [Ca^2+^]_i_, and increases in ROS, although the molecular mechanisms are not completely understood [42,43]. In an earlier study, it was reported that exposure of human bronchial epithelial cells to cigarette smoke induced ATP release that was reduced by inhibitors of pannexin 1 channels [44]. Furthermore, pannexin-1 deficient mice displayed significant inhibition of cigarette smoke-induced ATP release into the BAL. Our observation that pretreatment with GSH failed to inhibit pannexin-1 mediated ATP release suggests that a mechanism other than oxidative stress is responsible for PEI-induced increases in pannexin-1 activity. This result is consistent with previous findings where oxidative stress induced by Alternaria did not activate pannexn-1 dependent ATP efflux [26,41]. From the results of the present study, we conclude that PEI and Alternaria activate a common pathway for ATP release involving VDAC-1, however, unlike Alternaria, PEI does not induce vesicular ATP release [28]. 

Another important similarity between the epithelial response to PEI and allergens from Alternaria and HDM is the increase in [Ca^2+^]_i_ in response to ATP release [28,40]. However, the PEI-evoked Ca^2+^ response was more rapid than observed with Alternaria, and more sustained than the transient increase induced by HDM extract. Inhibition of ATP release using blockers of pannexin 1 or VDAC-1 also significantly inhibited PEI-induced increases in [Ca^2+^]_i_, providing evidence of an underlying role for purinergic signaling in regulation of the PEI response. Similarly, reducing ROS levels with GSH nearly abolished the PEI-stimulated increase in [Ca^2+^]_i_, indicating that, like *Alternaria*, inhibition of ATP release by reducing oxidative stress also blocks the increase in [Ca^2+^]_i_. Removal of extracellular Ca^2+^ completely inhibited the effect of PEI on [Ca^2+^]_i_, demonstrating that Ca^2+^ uptake was responsible for increasing [Ca^2+^]_i_, as previously shown for Alterneria [27]. Two Ca^2+^ uptake mechanisms were shown to be involved in the PEI response, one that was blocked by oATP and another that was inhibited by the potent (IC_50_ = 25 nM) and selective TRPV1 antagonist A784168 [45]. When both uptake pathways were blocked, no increase in [Ca^2+^]_i_ was produced following PEI exposure, demonstrating that PEI-stimulated Ca^2+^ uptake can be completely accounted for by activation of oATP and A784168 sensitive pathways. In an earlier study, the increase in [Ca^2+^]_i_ evoked by Alternaria extract was completely blocked by oATP, which was shown to be the result of P2X receptor inhibition [27]. Interestingly, A784168 failed to block the Alternaria-stimulated increase in [Ca^2+^]_i_ suggesting that TRPV1 does not appear to be not involved in mediating Ca^2+^ uptake induced by Alternaria extract [46]. 

In addition to transporting metal cations such as Na^+^ and Ca^2+^, TRPV1 receptors and several P2X receptor subtypes also conduct large organic cations including YoPro-1, rhodamine, 4,6-diamidino-2-phenylindole (DAPI), ethidium bromide, and N-methyl-D-glucamine (NMDG) [47,48,49,50]. In the present study, we showed that both PEI and Alternaria stimulate YoPro-1 and rhodamine influx across the plasma membrane. The PEI-dependent increase in cationic dye uptake appeared to be linked to ATP release since YoPro-1 uptake was completely blocked by oATP. Previous studies have shown that oATP is capable of inhibiting multiple P2X receptor subtypes (P2X_1_, P2X_2_, P2X_7_) known to transport organic cations, but not TRPV1 receptors or P2Y_2_ receptors [51,52,53]. The exact mechanism by which PEI activates TRPV1 receptors is presently unclear. Previous studies with silica nanoparticles (SiNPs) have suggested that cell surface perturbations resulting from SiNP particles striking the plasma membrane can produce TRPV1 activation by mechanical stimulation [54]. In contrast, insoluble electrophilic compounds present in coal fly ash particles activate TRPV1 through interactions with amino acids within the pore-loop region of the channel [55]. Diesel exhaust particles can also open TRPV1 channels in epithelial cells by indirectly activating protease-activated receptor 2 (PAR2), resulting in Ca^2+^ mobilization from internal stores, inflammatory mediator release and matrix metalloprotease activation [54]. Increasing extracellular concentrations of Na^+^, Mg^2+^, and Ca^2+^ also opens TRPV1 channels expressed in HEK293 cells and oocytes presumably by interacting with two glutamate residues (E600 and E648) located near the pore region of the channel [56]. Similarly, charge-dependent TRPV1 activation occurs following exposure to polyamines including spermine and putrescine [57]. These findings agree with earlier results showing that eosinophil granule proteins activate pulmonary sensory nerves that express TRPV1 channels [58]. Thus, it appears plausible that positively charged PEI nanoparticles also interact electrostatically with TRPV1 to produce channel opening and uptake of Ca^2+^; however, activation by this mechanism does not appear to facilitate conduction of large cations such as YoPro-1.

In addition to stimulating ATP release and increasing both intracellular [Ca^2+^] and organic cation uptake, PEI exposure produced DNA fragmentation. Inhibiting PEI-induced oxidative stress with GSH significantly reduced fragmentation, as did inhibition of ATP release and Ca^2+^ uptake. Genotoxic effects of linear and branched PEI polymers were investigated previously using a human squamous carcinoma cell line (A431 cells). Branched, but not linear PEI polymers, induced some tail formation in comet assays, indicating DNA fragmentation [59]. Moreover, DNA damage resulting from double-stranded breaks (DSBs) was also caused by exposing human bronchial epithelial cells (BEAS-2B cells) to HDM allergens [60,61]. HDM increased oxidative damage to proteins, lipids and nucleic acids (8-oxyguanine), reduced cell proliferation, and caused cell death. GSH and catalase prevented DNA fragmentation suggesting a role for oxidative stress in genotoxicity. Interestingly, DNA fragmentation was measured after 6 h of exposure to HDM, whereas in the present study, PEI-induced DNA fragmentation was detected after exposure for only 30 min [60]. As observed with HDM, oxidative stress appeared to be involved in producing DNA fragmentation. However in contrast to HDM, direct nucleotide oxidation to form 8-oxyguanine does not seem to be involved in PEI-induced fragmentation. This speculation is based on the observation that inhibition of ATP release and increases in intracellular [Ca^2+^] have inhibitory effects on DNA damage that are similar to blocking oxidative stress with GSH. Given that the increase in [Ca^2+^]_i_ is downstream of PEI-induced oxidative stress, it seems likely that raising intracellular [Ca^2+^] may stimulate DNase activity, perhaps by activating caspase 3. Further experiments will be required to determine whether increased caspase 3 activity is involved in mediating the effects of PEI on DNA fragmentation.

PEI and Alternaria also stimulated IL-33 mRNA expression. Moreover, oxidant scavenging with GSH along with inhibiting increases in [Ca^2+^]_i_ or blocking ATP release reduced the response. The increase in mRNA expression was associated with a concentration-dependent increase in IL-33 secretion into the extracellular fluid, which was also inhibited by the same pretreatment conditions that reduced IL-33 mRNA expression and similar to results of previous studies showing that Alternaria stimulates IL-33 secretion [26,27,34,62,63]. In addition, in vivo experiments showed that intranasal administration of PEI stimulated IL-33 secretion into the BAL fluid. Increased expression and secretion of IL-33 suggested that PEI was capable of inducing type-2 immunity. This conjecture was confirmed in subsequent in vivo studies where increases in the levels of Th2 cytokines, (IL-5 and IL-13) and the cytokine alarmin IL-33 were detected in the BAL fluid of mice exposed to PEI alone. Similarly, increases in BAL fluid [IL-5] and [IL-13] in mice treated with PEI/ovalbumin polyplexes were reduced in ST2 deficient mice. Furthermore, PEI/ovalbumin-induced increases in the number of eosinophils within the BAL fluid were also lower in ST2 knockout mice. These findings support the conclusion that IL-33 release induced by PEI, stimulated type 2 immune responses in vivo. They are also consistent with a previous in vivo study showing that PEI alone activated genes involved in Th1 and Th2 immunity in spleen lymphocytes and that the response was enhanced when PEI was formulated with DNA [64]. More recently, PEI was shown to exhibit robust mucosal adjuvanticity and protective immunity against influenza and herpes simplex virus-2 when administered intranasally with hemagglutinin or glycoprotein D antigens co-formulated with PEI [65]. Moreover, when branched PEI was used for surface functionalization of a graphene oxide (GO) based vaccine delivery vector, enhanced interactions between GO and recombinant influenza hemagglutinin (HA) occurred that resulted in positively charged nanoparticles with mucosal adjuvant activity [66]. Intranasal administration of GO-HA nanoparticles, in the absence of any additional adjuvants, stimulated robust, antigen specific immune responses that were protective against homologous and heterologous influenza viruses.

## 4. Materials and Methods

### 4.1. Materials

Alternaria (Alternaria alternata) and house dust mite (Dermatophagoides pteronyssinus) were purchased from Greer Laboratories (Lenoir, NC, USA). Polyethylenimine (PEI), 4,4′-diisothiocyanatostilbene-2,2′-disulfonic acid disodium salt hydrate (DIDS), L-glutathione (GSH), ATP, adenosine 5′-triphosphate, periodate oxidized sodium salt (oATP), probenecid, rhodamine B, ovalbumin and NaOH were purchased from Sigma-Aldrich Chemicals (St Louis, MO, USA). ^10^Panx, trovafloxacin mesylate (TVM), A784168 were purchased from Tocris (Minneapolis, MN, USA). ATP determination kit, Hanks’ balanced salt solution (HBSS), acetoxymethyl ester form of fura-2 (fura-2 AM), SYBR^®^ Gold, Yo Pro-1 and EDTA were obtained from ThermoFisher Scientific (Waltham, MA, USA).

### 4.2. Methods

#### Cell Culture Conditions

Human bronchial epithelial (hBE) cells were immortalized following transfection of genes encoding cyclin-dependent kinase-4 and human telomerase reverse transcriptase [67]. Cell monolayers were grown on two-well chamber slides (Laboratory-Tek, VWR International, Chicago, IL, USA) for Ca^2+^ imaging and dye uptake experiments, or on 35 mm culture dishes (Corning Life Sciences, Lowell, MA, USA) for ATP release measurements and for comet assays. The cells were cultured in bronchial epithelial cell growth medium with growth factor supplements (PromoCell GmbH, Heidelberg, Germany) and incubated at 37 °C in a humidified atmosphere of 5% CO_2_ in air.

### 4.3. ATP Release Measurements

ATP release into the medium was measured in real time using a luciferin/luciferase bioluminescence ATP determination kit. Cell monolayers were washed and replaced with 1 mL of standard reaction solution (SRS) containing luciferin/luciferase and loaded into a Glomax 20/20 luminometer (Promega, Madison, WI, USA). The background luminescence signal was measured for 1 min followed by PEI exposure and the change in luminescence was measured continuously for 10 min. The background luminescence signal was subtracted from the PEI-stimulated signal, then converted to [ATP] using an ATP calibration curve (range: 0.1–100 nM) and expressed as nM/cm^2^. The data points from 0.5–2.0 min after PEI exposure were used for linear regression analysis and the slope represented the initial rate of ATP release expressed as pmol/(min·cm^2^). 

### 4.4. Intracellular [Ca^2+^] Measurements

hBE cells grown on chamber slides (48–72 h), they were washed with HBSS containing 10 mM HEPES buffer and incubated with Fura-2 AM for 1 h. The cells were washed again and replaced with HBSS buffer, then mounted onto the stage of an inverted fluorescence microscope for measurements of intracellular Ca^2+^ concentration ([Ca^2+^]_i_). Fluorescence was measured using a Nikon UV 20× objective at excitation wavelengths of 340 nm/380 nm and an emission wavelength of 510 nm. MetaMorph software (Molecular Devices, San Jose, CA, USA) was used for image acquisition and data analysis. Relative changes in [Ca^2+^]_i_ were determined and expressed as the fluorescence ratio when the cells were excited at 340 nm and 380 nm (F340/F380).

### 4.5. Organic Cation Uptake Experiments

hBE cells were grown on two-well chamber slides for 48 h prior to use in dye uptake experiments. Culture media was replaced with 1 mL HBSS solution containing 10 mM HEPES, pH 7.4 and 2 μM Yo Pro-1. Chamber slides were then mounted onto the stage of a fluorescence microscope and images of the cells were acquired with a Prime 95B sCMOS digital camera (Teledyne Photometrics, Tucson, AZ, USA) using a 40× fluorescence objective (peak excitation/emission λ = 490/520 nm). Time course experiments were initiated with the addition of PEI (5 μg/mL) or Alternaria (100 μg/mL) or ATP (250 μM) and images of the cells were captured at 0.5 or 1.0 min intervals for 15 min and analyzed using Micro-Manager 1.4 software (https://micro-manager.org/ access date: 23 October 2017). A similar protocol was used for the rhodamine B uptake experiments, where cells were incubated in HBSS containing 0.5 μM rhodamine B for 10 min prior to addition of Alternaria (100 μg/mL) for 15 min. Images of the cells were acquired using a 20× fluorescence objective (peak excitation/emission λ = 545/570 nm).

### 4.6. Comet Assay

The effects of PEI on DNA fragmentation were examined using a CometAssay^®^ Kit (Trevigen, Gaithersburg, MD, USA). After PEI exposure, cells were trypsinized and combined with molten LM Agarose (37 °C) at a 1:10 *v*/*v* ratio. The combined cell-LM Agarose solution was pipetted onto comet slides and placed flat at 4 °C in the dark for 10 min, then slides were immersed in 4 °C lysis solution for 30–60 min to lyse the cells. To unwind and denature the DNA, the slides were immersed in alkaline electrophoresis solution (200 nM NaOH, 1 mM EDTA) for 20 min and then placed in an electrophoresis slide tray with a protective overlay on top. Gel electrophoresis was performed using the CometAssay^®^ ES unit, with 4 °C alkaline electrophoresis solution at 21V for 30 min. The slides were gently immersed twice in distilled H2O, then in 70% ethanol for 5 min each. After drying the slides at 37 °C for 10 min, SYBR^®^ Gold staining solution containing 10 mM Tris-HCl, 1 mM EDTA was placed onto the dried agarose and stained for 30 min in the dark. DNA fragmentation was visualized using an inverted fluorescence microscope with a Nikon 10x fluorescence objective at excitation/emission wavelengths of 496 nm/522 nm. Quantitative and statistical analyses were performed using CometScore 2.0 software (http://rexhoover.com/index.php?id=cometscore access date: 22 April 2021) and DNA fragmentation was expressed as percent DNA in the head and tail.

### 4.7. Quantitative Reverse-Transcription PCR (qRT-PCR) 

Total RNA was extracted from hBE cells transfected with the full-length human IL-33 gene (GET-33 cells) using RNeasy Mini Kit (Qiagen, Hilden Germany). One µg of total RNA quantified by Qubit Fluorometric assay (ThermoFisher, Waltham, MA, USA) was reverse transcribed to cDNA using a High Capacity RNA to cDNA kit (ThermoFisher). One µg cDNA, TaqMan probes (Hs00369211_m1, Hs02786624_g1), and TaqMan Fast Advanced Master Mix (ThermoFisher) were used to preform qRT-PCR on a Step-One-Plus Real-time PCR machine. The expression level was normalized to the threshold cycle number (Ct) of an internal reference gene (GAPDH).

### 4.8. In Vivo Experiments

Wild-type (WT) BALB/c mice were purchased from Jackson Laboratories. ST2^−/−^ (Il1rl1^−/−^) mice on a BALB/c background were kindly provided by Dr. Andrew N. McKenzie (Medical Research Council Laboratory of Molecular Biology, Cambridge, UK). All animal experiments were performed with the approval of and following the regulatory guidelines and standards set by the Institutional Animal Care and Use Committee of Mayo Clinic (protocol A59315, approved 18 November 2015). Female mice ages 6–13 weeks old were held under specific pathogen-free conditions prior to use in experiments. 

For acute PEI exposure, WT BALB/c mice were administered a single intranasal (i.n.) dose of phosphate buffered saline solution (PBS) with 12.5 µg PEI under isoflurane anesthesia. In chronic exposure experiments, WT BALB/c or ST2^−/−^ mice were intranasally challenged with 10 µg ovalbumin (OVA) in the absence of an adjuvant with or without 25 µg PEI following the treatment scheme below. After the final administration bronchoalveolar lavage (BAL) fluid was collected through a tracheostomy tube at 1 h after euthanasia with pentobarbital. Cell numbers in BAL fluid were counted using a hemocytometer. Eosinophils, neutrophils, lymphocytes, or macrophages were identified using standard morphologic criteria under light microscopy and percentages of these cells were determined.


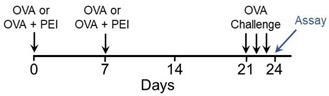


### 4.9. Cytokine Release Measurements

Measurement of IL-5, IL-13, and IL-33 concentrations in BAL fluid and GET-33 cell culture media or Hank’s buffered saline solution (HBSS) were performed using Quantikine or Duoset ELISA kits (R&D Systems, Minneapolis MN, USA), following the manufacturer’s instructions. Note that sensitivity for IL-5 was 8 pg/mL, for IL-13, 4 pg/mL and for IL-33, 14 pg/mL. Protein concentrations in the BAL fluid were quantitated using a BCA Protein Assay Kit (Bio-Rad Laboratories, Hercules, CA, USA).

### 4.10. IgE Assay

IgE measurements were performed following the protocol by Kobayashi et al., 2009 [68]. Briefly, ELISA plates (Immulon 4 HBX; Thermo Labsystems, Philadelphia, PA, USA) were coated with 5 μL/mL rat anti-mouse IgE mAb (LO-ME-3; Bio-Rad, Hercules, CA) in 0.1 M carbonate buffer (pH 9.5) for 2 h at 37 °C. Blocking was performed with PBS containing 1% BSA (MilliporeSigma, Burlington, MA, USA) overnight at 4 °C. After blocking, plasma samples diluted with PBS containing 1% BSA and 0.05% Tween 20 were added to the plates, and incubated for 2 h at room temperature. Afterwards, plates were treated with 1 μg/mL OVA or OVA + PEI, which had been biotinylated using a microbiotinylation kit (MilliporeSigma,), for 1 h at room temperature, followed by 1/5000 streptavidin-poly-HRP (ThermoFisher) for 30 min at room temperature. After the final washing, peroxidase substrate (TMB substrate kit) was added and the reaction stopped 20 min later with 1 M HCl. After each step, plates were washed with PBS containing 0.05% Tween 20. A microplate autoreader (Thermomax; Molecular Devices, San Jose, CA, USA) was used to measure absorbance at 450 nm.

### 4.11. Statistics

Data are presented as means ± standard error. Statistical comparisons between means from multiple treatment conditions were determined using a Brown-Forsythe and Welch one-way ANOVA followed by Dunnett’s T3 posttest or by a standard one-way ANOVA followed by Tukey’s multiple comparisons test. A two-tailed, unpaired t test was used for statistical comparisons between two means. Graphics and statistical analyses were performed using GraphPad PRISM 8.0 (San Diego, CA, USA).

## 5. Conclusions

The findings of the present study provide new insights into the mechanisms by which PEI induces type 2 immunity. The airway epithelial response to PEI exposure was similar to that of certain allergens that stimulate ATP release and subsequently increase intracellular [Ca^2+^]. Most of the ATP release occurred in response to oxidative stress and Ca^2+^ uptake was required for increasing IL-33 mRNA transcription and secretion into the extracellular media. These findings indicate that mucosal adjuvanticity associated with PEI alone or with PEI polyplexes requires early induction of ATP release as a means of promoting IL-33 mobilization and secretion from the airway epithelium. Subsequent interaction of IL-33 with ST2 receptors associated with ILC2 cells, Th2-type CD4+ T cells, and perhaps other inflammatory cells facilitates the production and release of Th2 cytokines like IL-5 and IL-13, which ultimately leads to development of an allergic inflammatory response.
